# Accuracy and Incremental Yield of the Chest X-Ray in Screening for Tuberculosis in Uganda: A Cross-Sectional Study

**DOI:** 10.1155/2021/6622809

**Published:** 2021-03-19

**Authors:** Joanitah Nalunjogi, Frank Mugabe, Irene Najjingo, Pastan Lusiba, Francis Olweny, Joseph Mubiru, Edward Kayongo, Rogers Sekibira, Achilles Katamba, Bruce Kirenga

**Affiliations:** ^1^Makerere University Lung Institute, College of Health Sciences, Makerere University, Uganda; ^2^Clinical Epidemiology Unit, College of Health Sciences, Makerere University, Uganda; ^3^Uganda National Tuberculosis and Leprosy Program, Ministry of Health, Uganda; ^4^Accident and Emergency Unit, Mulago National Referral Hospital, Kampala, Uganda; ^5^Division of Pulmonary Medicine, College of Health Sciences, Makerere University, Uganda

## Abstract

The WHO END TB strategy requires ≥90% case detection to combat tuberculosis (TB). Increased TB case detection requires a more sensitive and specific screening tool. Currently, the symptoms recommended for screening TB have been found to be suboptimal since up to 44% of individuals with TB are asymptomatic. The chest X-ray (CXR) as a screening tool for pulmonary TB was evaluated in this study, as well as its incremental yield in TB diagnosis using a cross-sectional study involving secondary analysis of data of 4512 consented/assented participants ≥15 years who participated in the Uganda National TB prevalence survey between 2014 and 2015. Participants with a cough ≥2 weeks, fever, weight loss, and night sweats screened positive for TB using the symptoms screening method, while participants with a TB defining abnormality on CXR screened positive for TB by the CXR screening method. The Löwenstein-Jensen (LJ) culture was used as a gold standard for TB diagnosis. The CXR had 93% sensitivity and 65% specificity compared to LJ culture results, while symptoms had 76% sensitivity and 31% specificity. The screening algorithm involving the CXR in addition to symptoms led to a 38% increment in the yield of diagnosed tuberculosis. The number needed to screen using the CXR and symptoms screening algorithm was 32 compared to 45 when the symptoms are used alone. Therefore, the CXR in combination with symptoms is a good TB screening tool and increases the yield of diagnosed TB.

## 1. Introduction

The WHO 2020 estimates show that tuberculosis (TB) was responsible for 1.4 million deaths [1]. In order to combat tuberculosis, WHO proposed three strategies including intensified case finding (ICF), isoniazid preventive therapy (IPT), and infection control (IC) [2]. In addition, the WHO END TB strategy indicates a need for ≥90% TB case detection among others to combat TB [3].

Uganda has a high TB incidence of 201 per 100,000 population as well as high TB-related mortality of 26 per 100,000 population with a TB case detection rate of 72% [4].

The strategic plan for the Uganda national TB and leprosy programme (NTLP) sought to achieve 85% case detection by 2019/2020; however, confirming TB was difficult. Currently, TB culture is the gold standard, but financial and logistic challenges make it difficult to scale up. There is a need to explore more widely available, low-cost screening and diagnostic tools and algorithms to aid TB diagnosis such as the chest radiograph (CXR), as improved TB diagnostics (Gene Xpert) have now become available. Currently, the symptoms screening tool is employed for TB screening in Uganda. This symptoms screening tool has been observed to have 40.7% sensitivity and 81.3% specificity according to a survey done in South Africa considering the presence of any cough, fever, weight loss, or night sweats [5]. Systematic reviews done to assess the performance of the symptoms in high HIV prevalence regions such as Uganda have shown 84% sensitivity and 74% specificity [6]. The symptoms screening tool therefore runs a risk of missing patients with TB as the patients do not seek care and do not have symptoms due to early TB disease. These patients if not diagnosed early by a more specific screening test will continue to spread TB in the community and present with complications due to TB hence poor treatment outcomes.

The recognition that bold new strategies are needed to control TB (END TB Strategy) has led to reconsideration of CXR as a potential TB screening tool, as innovative, lower costs strategies for performing CXR (digital radiography) and reading them (computer-aided reading) have become available [7, 8]. The CXR was commonly used in mass screening campaigns in Europe and the USA in the early TB chemotherapy era (1950s) but has not been widely used in screening TB in low-income countries because of high cost and lack of capacity [9, 10]. This CXR has been found to have 98% sensitivity and 75% specificity in a systematic review done among HIV-negative and HIV-positive individuals [6].

The use of more sensitive TB screening tools such as the CXR may increase the pool of presumptive TB cases hence increasing case detection as more individuals are exposed to the confirmatory test (Gene Xpert or Culture) which ultimately leads to a reduction in mortality and morbidity [11]. In order to assess the accuracy of the CXR in screening tuberculosis, there is a need to use a test known to confirm tuberculosis such as Lowenstein Jensen (LJ) culture.

Uganda carried out a national TB prevalence survey (UTPS) from 2014 to 2015 which screened for TB by collecting data on TB symptoms using questionnaires and performed CXR in all consenting participants; in addition, all sputa were cultured on LJ media to confirm tuberculosis [12].

Therefore, using the data from UTPS, we sought to evaluate the accuracy and incremental yield of using the CXR in screening for TB in Uganda.

## 2. Materials and Methods

### 2.1. Study Design

We conducted a cross-sectional study by secondary analysis of the data collected during the Uganda National TB prevalence survey (UTPS) [13]. The UTPS was conducted from October 2014 to July 2015 with the primary objective of determining the prevalence of TB in Uganda. Eligible respondents aged ≥15 years were randomly selected in blocks to participate in the study [12, 13].

All eligible consenting/assenting individuals who were ≥15years were screened for TB using a questionnaire which listed the symptoms and a CXR. Parents/guardians of individuals <18 years offered informed consent. Participants with a cough ≥2 weeks, TB defining abnormality on CXR examination/or any abnormality in the lung on CXR examination, and respondents without a CXR were considered presumptive for TB. All presumptive TB respondents were requested to submit two sputum samples (a spot and an early morning sample) for microscopy and culture. All positive samples by microscopy and contaminated culture samples were subjected to Xpert MTB/RIF test to confirm the presence of mycobacterium tuberculosis (MTB). The CXRs were read by 3 independent radiologists. LJ culture was used as a gold standard for the confirmation of tuberculosis among the survey participants. All records in the electronic database that had bacteriological confirmation of TB as per UTPS by culture were analysed for the substudy [12]. Participants with mycobacterium other than tuberculosis (MOTT) were identified using SD Bio line on positive cultures and Xpert MTB/RIF done on positive smears and contaminated cultures. All individuals with MOTT were not reported as MTB cases. In the UTPS, all cases of TB reported as survey TB were confirmed on culture.

The STARD 2015 guidelines [14] were used to report the results of this study. Data was analysed using Stata version 13.0 (College Station, Texas, USA). In our study, records with any of the above symptoms, i.e., cough ≥2 weeks, fever, weight loss, or night sweats were analysed as positive for symptoms. Records indicating CXR abnormalities suggestive of active TB were analysed as positive by the CXR. Records with culture-confirmed TB were considered as the gold standard for determining the performance of the CXR or symptoms in screening tuberculosis.

Variables were summarised using percentages. Performance of the symptoms screening and CXR were reported as sensitivity, specificity, and negative and positive predictive values. The proportion of confirmed TB cases obtained using the CXR screen was reported as the yield due to the CXR in percentages. The proportion of confirmed TB cases obtained using the symptoms screen was reported as yield due to the symptoms screen and reported in percentages. The incremental yield of the CXR was reported as a percentage increase in the total number of TB cases diagnosed through an algorithm involving the CXR versus an algorithm involving the symptoms screen alone.

## 3. Results

### 3.1. Study Profile

**Figure 1:**
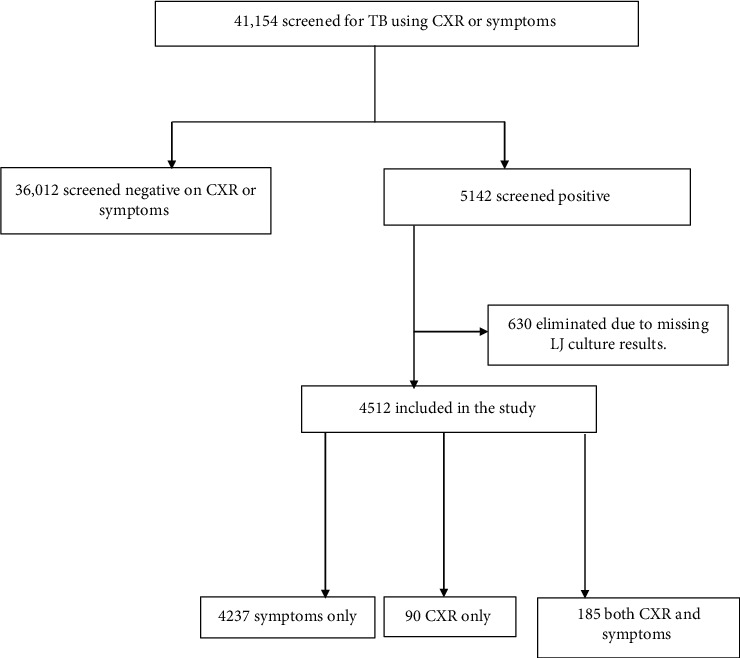
Study profile.

A total of 41,154 participants were enumerated for screening in the UTPS. We excluded 36,012 records of participants who screened negative on the CXR and symptoms and therefore were not required to submit sputum for TB diagnosis. 630 records that were missing data on culture results were additionally excluded leaving 4512 records for further analysis as shown below (Figure 1).

### 3.2. Characteristics of the Study Population

The study included individuals aged 15–65 years. The participants between ages 25 and 34 years were 846 (19%) as well as those in the age group 35–44 years 860 (19%). There was an equal number of females 2266 (50%) and males. Cough was present in 63% of the respondents, while 6% of the respondents had CXR abnormalities consistent with active TB disease. MOTT was present in 1% of the cultures (Table 1).

### 3.3. Performance of the Chest Radiograph or Symptoms in Screening TB in Uganda

Pulmonary TB was confirmed in 160 (3.6%) participants using LJ culture. The CXR had sensitivity 93% (95% CI; 87, 96) and specificity 97% (95% CI; 96, 98) compared to symptoms with sensitivity 76 (95% CI; 69, 83) and specificity 31% (95% CI; 30, 32).

The negative and positive predictive values of the CXR were 99% and 54% versus 97% and 4% of the symptoms. The CXR and symptoms combined had 100% sensitivity and 29% specificity. The positive and negative predictive values of this screening algorithm were 5% and 100%, respectively (Table 2).

### 3.4. Incremental Yield of Using the CXR in Screening TB versus Symptoms Alone

Using the symptoms alone in screening tuberculosis, 122/160 TB patients would have been diagnosed. The addition of the CXR in the TB screening algorithm increased the cases of TB diagnosed by 38 (23.8%). The number needed to screen was 32 individuals. The detailed results are shown in Table 3, below.

## 4. Discussion

This study compared the accuracy of the CXR or symptoms against culture-confirmed pulmonary TB by the Lowenstein Jensen (LJ) method in a community TB screening setting such as the National TB prevalence survey.

Our results show that the CXR performs better than the symptoms in correlating with positive TB sputum culture results in community settings just like other studies [2, 15–19]. The sensitivity of 93% and specificity of 97% obtained when considering TB defining CXR abnormalities are comparable to the results obtained in a study done in London when a digital CXR was employed in active case finding [18]. These results indicate that the CXR performs better in correlating TB sputum culture results; therefore, the use of the CXR in addition to symptoms in TB screening in community settings increases TB case detection rate [20] in high TB prevalence settings which allows for timely initiation of TB treatment [19] as bacteriological confirmation and drug susceptibility tests are processed to further determine appropriate TB treatment [19]. Additionally, our screening algorithm involving both the CXR and symptoms showed 100% sensitivity and 29% specificity which results differ from those of a systematic review of TB screening algorithms where a screening algorithm involving both the CXR and symptoms showed sensitivity 87% and specificity 90% [6]. This difference could be explained by the fact that only individuals with symptoms and abnormal CXR exam were eligible for sputum evaluation in the UTPS hence overestimation of the sensitivity at the cost of specificity. However, the use of the CXR increases the overall sensitivity hence an increased number of individuals being subjected to the confirmatory tests resulting into increased TB yield [21, 22]. This is especially important in the era of the new advanced TB diagnostic tools such as the GeneXpert with high sensitivity and specificity [23].

In our study, the symptoms screen showed a sensitivity of 76% and specificity of 31%. These estimates are similar to those obtained in a systematic review of TB screening algorithms [6]; however, studies done in Asia, Zambia, and South Africa to evaluate the accuracy of symptoms in screening TB showed slightly different estimates of sensitivity and specificity of symptoms compared to those seen in our study [24, 25]. This could have resulted from the difference in the population under study, since our study only had cultures done from patients who screened positive by CXR or symptoms or did not have a CXR done.

Furthermore, a 38% increment in the number of TB cases diagnosed as well as a low number needed to screen 32 patients when using a screening algorithm involving both the CXR and symptoms is a good step towards interventions leading to increased TB case finding hence TB elimination. This increment was however lower than that reported in a study done in India among household contacts of TB patients [21]. The difference may be explained by the special population under which the study in India was done which is a more high-risk population for TB compared to the general population that we studied as well as the higher prevalence of TB in India compared to Uganda. This finding however provides insight into the innovations that may lead to the achievement of the 90% TB detection for the END TB strategy [3]. The results are also in agreement with a study done in South Africa on symptoms and digital CXR screening which recorded improvement in the yield of TB [26] screening among prisoners when both screening strategies were employed. The addition of the CXR to symptoms in screening TB can be employed to identify potential TB cases that would be missed when using only symptoms for screening. From our study, the algorithm involving the symptoms screening alone would have missed 23% of the patients with TB. Our finding is also supported by earlier reports of similar studies from different TB endemic countries [5, 13, 17, 24]. The strengths of our study include community screening for TB as shown by the use of data collected during the UTPS. The study was reported according to the STARD (Standards for reporting of Diagnostics Accuracy Studies) 2015 guidelines [14].

Our study is however subject to limitations. The fact that sputum culture was only done for participants with abnormal CXR or TB symptoms could have led to selection bias hence overestimation of the sensitivity of the CXR; however, the large sample size improves our estimation of the performance of the CXR. In addition, we only looked at culture-confirmed pulmonary TB but not clinically diagnosed and not culture-negative or extrapulmonary TB which could greatly impact the performance of symptoms screening and/or CXR to identify true TB. Additionally, the failure to use more sensitive TB confirmatory tests such as the Gene Xpert and Liquid culture could have led to underestimation of confirmed TB hence lead to underestimation of the performance of the CXR or symptoms. Finally, the study was performed under community screening conditions, and therefore, the results may not be applicable to hospital-based TB screening.

## 5. Conclusion

This study made use of data obtained during the Uganda National TB prevalence survey to determine the performance of the CXR in correlating TB culture results. The results indicate that the CXR is a good TB screening tool and may improve TB case detection when used in community TB screening in addition to symptoms assessment. Individuals with knowledge to observe TB defining pathology on the CXR are adequate to provide expertise for CXR interpretation.

## Figures and Tables

**Table 1:** Demographic, clinical, and radiographic characteristics of the study population.

Characteristic	*N* = 4512 N (%)
Age	
15-24 yrs	767 (17)
25-34 yrs	846 (19)
35-44 yrs	860 (19)
45-54 yrs	727 (16)
55-64 yrs	474 (11)
65+ yrs	838 (18)
Female	2266 (50)
Education	
None	1283 (28)
Primary	2135 (47)
Secondary	890 (20)
Tertiary	204 (5)
Employed	3603 (80)
Rural residence	2889 (64)
Smoking	
Never	3277 (73)
Current	640 (14)
Past	595 (13)
HIV^∗^	
Positive	392 (10)
Symptom characteristics^∞^	
Cough^∞^	2827 (63)
Fever^∞^	668 (14)
Weight loss^∞^	608 (14)
Night sweats^∞^	394 (9)
CXR characteristics^∗∗^	
Normal	2784 (63)
Abnormal healed TB	232 (5)
Abnormal active TB	275 (6)
Abnormal not TB	1150 (26)
Culture confirmed TB	
MTB	160 (3)
MOTT	26 (1)
Contaminated	57 (1)
Negative	4269 (95)

^∗^missing 474/4512 (10.1%), ^∗∗^missing 71/4512 (1.6%) participants who declined to have a CXR examination, ^∞^each individual characteristics *N* = 4512.

**Table 2:** Performance of the CXR or symptoms in screening culture-confirmed pulmonary tuberculosis.

Strategy	Culture-confirmed TB	Total number	Sensitivity (95% CI)	Specificity (95% CI)	Positive predictive value (95% CI)	Negative predictive value (95% CI)
Prevalence	160	4512				
CXR	148	4441	93 (87, 96)	97 (96, 98)	54 (48, 60)	99 (99, 100)
Symptoms	122	4512	76 (69, 83)	31 (30, 32)	4 (3, 5)	97 (96, 98)
CXR+symptoms	160	4512	100 (98, 100)	29 (28, 31)	5 (4, 6)	100 (99, 100)

**Table 3:** Incremental yield and number needed to screen of using the CXR in a TB screening algorithm.

Strategy/algorithm	Screened	Tested	NNS	Number of TB cases	% of confirmed TB	Incremental yield (%)
Symptoms only	5142	4512	42	122	76	Ref
X-ray only	5142	4441	35	149	93	17
Symptoms and X-ray	5142	4441	32	160	100	23

NNS: number needed to screen (number of people needed to screen to diagnose a bacteriologically positive case, who has not been previously diagnosed by the health system). TB: tuberculosis.

## Data Availability

All data generated or analysed during this study are accessible on reasonable request from the corresponding author.

## Abbreviations

CXR: Chest X-ray
HIV: Human immune virus
IC: Infection control
ICF: Intensified case finding
IPT: Isoniazid preventive therapy
LJ: Lowenstein Jensen
MoH: Ministry of Health
MOOT: Mycobacterium other than TB
NNS: Number needed to screen
NTLP: National TB and leprosy programme
PLHIV: People living with HIV
PTB: Pulmonary tuberculosis
SOMREC: School of Medicine Research and Ethics Committee
TB: Tuberculosis
UTPS: Uganda TB prevalence survey
WHO: World Health Organisation.
